# Hepatocyte-Targeted Expression by Integrase-Defective Lentiviral Vectors Induces Antigen-Specific Tolerance in Mice with Low Genotoxic Risk

**DOI:** 10.1002/hep.24230

**Published:** 2011-05

**Authors:** Janka Mátrai, Alessio Cantore, Cynthia C Bartholomae, Andrea Annoni, Wei Wang, Abel Acosta-Sanchez, Ermira Samara-Kuko, Liesbeth De Waele, Ling Ma, Pietro Genovese, Martina Damo, Anne Arens, Kevin Goudy, Timothy C Nichols, Christof von Kalle, Marinee K L Chuah, Maria Grazia Roncarolo, Manfred Schmidt, Thierry VandenDriessche, Luigi Naldini

**Affiliations:** 1 Free University of Brussels, Brussels, Belgium; Vesalius Research Center, Flanders Institute of Biotechnology; University of LeuvenLeuven, Belgium; 2 San Raffaele Telethon Institute for Gene Therapy, San Raffaele Scientific InstituteMilan, Italy; 3 Vita Salute San Raffaele UniversityMilan, Italy; 4 National Center for Tumor Diseases, Department of Translational Oncology, German Research Cancer CenterHeidelberg, Germany; 5 University of North CarolinaChapel Hill, NC

## Abstract

Lentiviral vectors are attractive tools for liver-directed gene therapy because of their capacity for stable gene expression and the lack of preexisting immunity in most human subjects. However, the use of integrating vectors may raise some concerns about the potential risk of insertional mutagenesis. Here we investigated liver gene transfer by integrase-defective lentiviral vectors (IDLVs) containing an inactivating mutation in the integrase (D64V). Hepatocyte-targeted expression using IDLVs resulted in the sustained and robust induction of immune tolerance to both intracellular and secreted proteins, despite the reduced transgene expression levels in comparison with their integrase-competent vector counterparts. IDLV-mediated and hepatocyte-targeted coagulation factor IX (FIX) expression prevented the induction of neutralizing antibodies to FIX even after antigen rechallenge in hemophilia B mice and accounted for relatively prolonged therapeutic FIX expression levels. Upon the delivery of intracellular model antigens, hepatocyte-targeted IDLVs induced transgene-specific regulatory T cells that contributed to the observed immune tolerance. Deep sequencing of IDLV-transduced livers showed only rare genomic integrations that had no preference for gene coding regions and occurred mostly by a mechanism inconsistent with residual integrase activity. *Conclusion:* IDLVs provide an attractive platform for the tolerogenic expression of intracellular or secreted proteins in the liver with a substantially reduced risk of insertional mutagenesis. (hepatology 2011;)

Liver-directed gene therapy has the potential to treat metabolic diseases and plasma protein deficiencies, including hemophilia.^1^–^3^ Moreover, hepatic gene transfer can favor the induction of immune tolerance, so it may also be considered for the prevention or treatment of autoimmune diseases.^4^,^5^ Because there is no need to overcome preexisting cellular or humoral immunity to viral vector components, the use of lentiviral vectors (LVs) represents a promising approach for liver gene transfer and immune tolerance induction.^6^–^8^

In previous studies, we showed that immune tolerance induction with LVs is dependent on stringent targeting of transgene expression to hepatocytes, which can be achieved by a combination of transcriptional and posttranscriptional microRNA-based regulation.^6^,^8^ Transcriptional control elements derived from hepatocyte-specific genes were incorporated into the vector design to preferentially express the transgene in hepatocytes. Because LVs readily transduce professional antigen-presenting cells,^9^ target sequences for a hematopoietic-specific microRNA—microRNA 142 (miR-142)–were incorporated downstream of the transgene to suppress any residual expression in antigen-presenting cells.^7^ This stringent regulation suppressed the induction of immunity against the transgene product in the injected mice and induced antigen-specific immunological tolerance, which could not be reversed by vaccination.^6^ Using this strategy, we also obtained long-term coagulation factor IX (FIX) expression in hemophilia B mice, which resulted in immune tolerance to FIX and correction of the disease phenotype.^8^

Although these studies have demonstrated the feasibility of gene replacement therapy and immune tolerance induction with LVs, concerns remain about the potential long-term adverse effects of vector integration due to insertional mutagenesis. Although advanced LV design potentially reduces this risk,^10^,^11^ the impact of LV integration in the liver is largely unexplored. Moreover, it is not known whether the tolerogenic outcome of microRNA-regulated LV delivery depends on sustained high levels of transgene expression within hepatocytes, which requires substantial levels of LV integration and would limit the application of this finding outside gene replacement strategies for the correction of monogenic diseases.

Integrase-defective lentiviral vectors (IDLVs) are typically generated by the packaging of the vector with catalytically inactive human immunodeficiency virus (HIV) integrase.^12^,^13^ The class I D64V mutation in the integrase catalytic site substantially reduces integration (10^2^- to 10^3^-fold) without compromising other steps in the transduction pathway and was thus adopted for this study.^12^,^14-18^ Upon transduction, IDLVs can support transgene expression from the nonintegrated proviral forms. Because this episomal DNA is progressively lost in actively dividing cells, transgene expression is only transient.^14^,^15^,^19^ In contrast, IDLVs have been reported to support sustained transgene expression in quiescent mouse tissues, such as the retina and central nervous system, likely because the episomal vector DNA is retained within the postmitotic nucleus.^17^,^18^,^20^ Despite these advances, the ability of IDLVs to achieve therapeutically meaningful transgene expression after hepatic gene delivery and to induce immune tolerance has never been studied. Moreover, the nature of any persistent IDLV genome in transduced cells is poorly defined because a comprehensive characterization of the residual IDLV integration profile is lacking. This study addresses all these outstanding questions and provides evidence supporting IDLVs as an emerging platform technology for liver gene transfer and immune tolerance induction.

## Materials and Methods

### Vector Construction

Plasmids pCCLsin.cPPT.ET. cFIX.142T, pCCLsin.cPPT.PGK.OVA, and pCC Lsin.cPPT.ET.OVA.142T (ET = enhanced transthyretin, OVA = ovalbumin, and PGK = phosphoglycerokinase) were constructed with standard cloning techniques. Details are available upon request.

### Mouse Experiments

Green fluorescent protein (GFP)–expressing integrase-competent lentiviral vectors (ICLVs) and IDLVs were administered to 7-week-old female BALB/c mice by tail vein injection. Six-week-old female and male C57BL/6 FIX-knockout mice were injected intravenously on 2 consecutive days with a total p24 dose of 260 μg (2 × 500 μL) in FIX-expressing ICLVs or IDLVs supplemented with 40 mg/mL polybrene. For 70% partial hepatectomy, mice were anesthetized with isoflurane. Liver sections were snap-frozen for genomic DNA extraction (Qiagen DNA extraction kit, Qiagen, Belgium). FIX activity in citrated plasma was quantified (Biophen factor IX chromogenic activity assay, Hyphen Biomed, France) according to the manufacturer's instructions with normal dog plasma as a reference (detection limit > 0.1%). All animal experiments were approved by the Animal Ethics Committee of the University of Leuven or the San Raffaele Institutional Animal Care and Use Committee (321).

### Integration Site (IS) Analysis

To analyze ICLV and IDLV integration, we used standard and nonrestrictive, 5′-long terminal repeat (5′-LTR)–mediated and 3′-LTR–mediated linear amplification–mediated polymerase chain reaction (LAM-PCR) as previously described.^21^,^22^

Further details can be found in the Supporting Information.

## Results

### IDLVs Efficiently Transfer Episomal Genomes Into Human Hepatocytes *In Vitro* but Drive Lower Levels of Transgene Expression Than Their Integration-Competent Counterparts

**Fig. 1:**
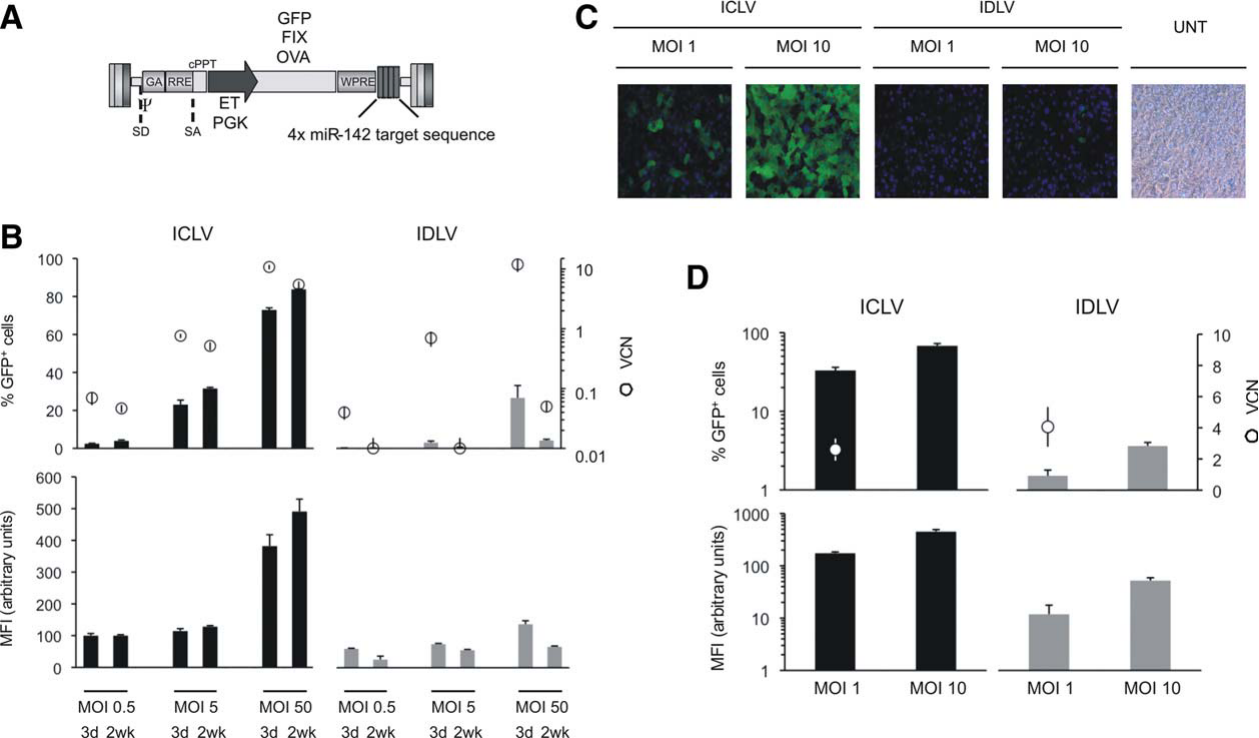
IDLV performance in hepatocytes culture (A) Schematic representation of the third-generation self-inactivating vector used for these studies. SD: splicing donor site. SA: splicing acceptor site. ψ: packaging signal (including 5' portion of GAG gene (GA)). RRE: Rev responsive element. cPPT: central polypurine tract. Wpre: woodchuck hepatitis virus post-regulatory elements. 142T: miR-142 target sequence made of 4 tandem copies of a sequence perfectly complementary to miR-142. Vectors were produced with integrase-competent (ICLV) or integrase-defective (IDLV) construct. The green fluorescent protein (GFP), coagulation Factor IX (FIX) and ovalbumin (OVA) were driven from the hepatocyte-specific ET promoter composed of synthetic hepatocyte-specific enhancers and transthyretin promoter or the ubiquitously expressed phosphoglycerokinase (PGK) promoter (B) Percentage of GFP^+^ cells and mean fluorescence intensity of GFP (MFI; left axis) and vector copies/diploid genome (vector copy number – VCN; right axis) in Huh7 cells transduced with ICLV or IDLV at the indicated multiplicity of infection (MOI) and analyzed 3 days or 2 weeks after transduction by flow cytometry. Black bars correspond to ICLV-transduced cells, grey bars to IDLV-transduced cells. Circles show VCN. The results are presented as mean ± standard error of the mean (SEM; n = 3). (C) Representative images of human primary hepatocytes transduced as indicated or left untreated (UNT) and analyzed by live fluorescence microscopy 1 week after transduction. Nuclei are stained with Hoechst. (D) Percentage of GFP^+^ cells and MFI of GFP (5 fields per sample; left axis) and VCN (circles, right axis) in cultures from quiescent human primary hepatocytes. The results are presented as mean ± range (n = 2). Abbreviations: MOI, multiplicity of infection; UNT, untreated.

We generated GFP-expressing LVs with either integrase-defective (IDLV) or integrase-competent (ICLV) packaging constructs and compared their transduction efficiency and transgene expression levels in human cell lines and primary hepatocytes *in vitro*. To drive transgene expression, we used a hepatocyte-specific chimeric promoter (designated as ET).^8^ The vectors carried target sequences for miR-142 in the transgene 3′-untranslated region (3′-UTR; ET.GFP.142T; Fig. 1A). The physical particle content was determined by HIV-1 group-specific antigen (Gag) p24 quantification. To determine infectivity, which we defined as transducing units per physical particle, we designed an *ad hoc* quantitative polymerase chain reaction (PCR) that selectively amplified the reverse-transcribed vector genome and discriminated it from plasmid DNA, which was carried over from the transfection used to produce the vectors (Supporting Information Fig. 1). IDLVs and ICLVs had similar infectivity in several human cell lines (Supporting Information Table 1 and data not shown). We transduced the hepatocyte Huh7 cell line and measured vector genomes and GFP expression at 3 days and 2 weeks post-transduction (Fig. 1B). Initially, comparable amounts of reverse-transcribed vectors were present in IDLV- and ICLV-transduced cells. However, the frequency of GFP^+^ cells and the mean fluorescent intensity (MFI) of GFP were lower in IDLV-transduced cells versus ICLV-transduced cells (*P* < 0.01, n = 3), and this indicated less efficient expression from the former vector. Analysis of the same cultures 2 weeks after transduction showed a nearly complete loss of vector genomes and GFP^+^ cells in the IDLV-transduced cells, as expected for an episomal form.

We then tested the same vectors on human primary hepatocytes, which do not proliferate in culture, and showed that IDLVs attained similar levels of transduced vector genomes vector copy number (VCN) 1 week after transduction but expressed GFP at substantially lower levels in comparison with matched doses of ICLVs (n = 2; Fig. 1C,D). Overall, these data indicate that IDLVs efficiently transfer episomal vector genomes into hepatocytes. However, these forms provide a less proficient substrate for the transcriptional machinery than the integrated proviral vectors.

### IDLVs Support FIX Expression From Hepatocytes in Mice

**Fig. 2:**
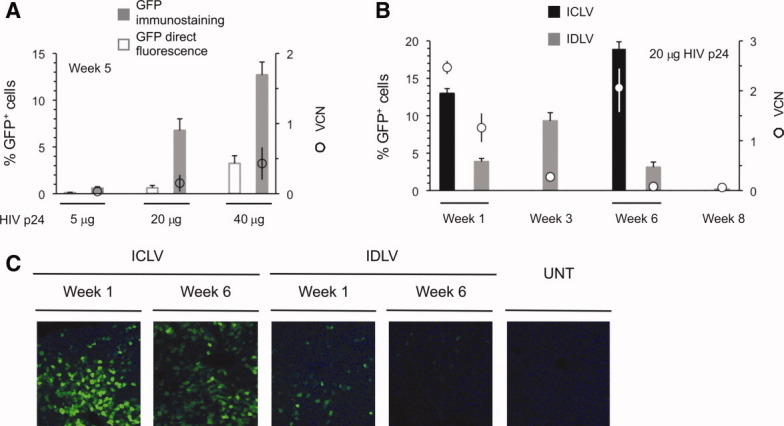
IDLV performance in the mouse liver (A) Morphometric analysis of GFP-expressing hepatocytes and VCN in the liver of mice injected with the indicated HIV-1 p24 Gag equivalents of IDLV.ET.GFP.142T and analyzed at the indicated time post-injection. GFP^+^ cells were identified either by immunostaining or direct fluorescence. Results are presented as mean ± SEM of n = 3 mice per time point, 5-10 optical fields scored from 5-10 non-consecutive GFP-immunostained (filled bars) or unstained (open bars) liver sections per mouse (left axis). Circles show VCN (right axis), as mean ± SEM. (B) Same analysis performed for IDLV- (grey bars) vs. ICLV- (black bars) injected mice, n = 3-7 mice per IDLV time point from 3 different experiments. (C) Representative images of unstained liver sections from mice reported in (B). Nuclei were stained with TOPRO-3. Abbreviation: UNT, untreated.

To ascertain that IDLVs could be used to transduce hepatocytes *in vivo*, we injected increasing doses (5-, 20-, and 40-μg HIV-1 Gag p24 equivalents, n = 8) of GFP-expressing IDLVs (ET.GFP.142T) intravenously into adult mice and measured GFP expression in hepatocytes and vector DNA contents in the liver 5 weeks post-injection (Fig. 2A). A vector dose–dependent increase in hepatic transduction was apparent; the yield of GFP^+^ hepatocytes in the treated livers was as high as 13% according to an analysis by GFP-specific immunostaining. Subsequently, we administered matched doses (20-μg HIV-1 Gag p24 equivalents) of GFP-expressing IDLVs and ICLVs (ET.GFP.142T) intravenously to adult mice (n = 20 for IDLV mice and n = 4 for ICLV mice in three independent experiments) and measured GFP expression and vector DNA contents in the liver at different times post-injection (Fig. 2B,C). One week post-injection, GFP-expressing hepatocytes were readily detectable in IDLV-treated mice, although the frequency and the intensity were substantially lower than those observed in mice treated with matched ICLV doses. The contents of reverse-transcribed vector genomes were only slightly lower in mice treated with IDLVs versus mice treated with ICLVs and reached up to 1.5 vector copies per diploid genome. However, although the GFP expression and the vector content remained stable in the ICLV-treated mice, the frequency of GFP^+^ hepatocytes and the vector content progressively decreased with increasingly longer times post-injection for the IDLV-treated groups.

**Fig. 3:**
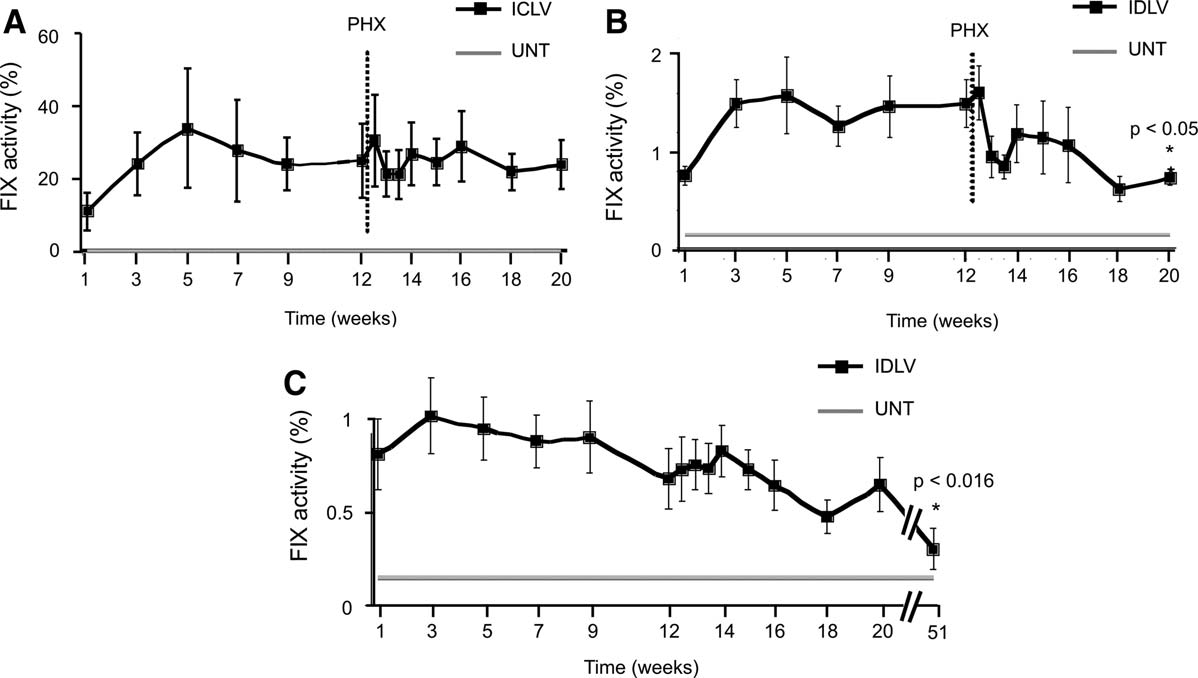
Functional FIX expression in hemophilic mice FIX KO mice were injected with ICLV.ET.FIX.142T (A) or IDLV.ET. FIX.142T (B) and FIX activity was monitored in the mouse plasma collected by retro-orbital bleeding. Partial hepatectomy (PHX) was performed at week 12. In both panels, grey lines correspond to untreated (UNT) FIX KO mice. (C) FIX activity in FIX KO mice injected with IDLV.ET.FIX.142T monitored for 1 year post injection and not subjected to partial hepatectomy. Abbreviations: PHX, partial hepatectomy; UNT, untreated.

We then administered matched high doses (260-μg HIV Gag p24 equivalents per mouse) of IDLV- or ICLV-expressing canine FIX complementary DNA (ET.FIX.142T) to adult hemophilia B mice (n = 15 for IDLV mice and n = 8 for ICLV mice; Fig. 3). IDLV delivery resulted in prolonged production of FIX in mouse plasma at levels considered within the therapeutic range (up to 1.5% of normal levels). In agreement with the different efficiencies of expression observed for the GFP marker, the FIX levels supported by IDLVs were up to 15-fold lower than those supported by the cognate ICLVs. Twelve weeks post-injection, the VCN in transduced livers was 0.22 ± 0.16 per diploid genome for the IDLV group and 4.36 ± 0.24 per diploid genome for the ICLV group. At this time, some of the IDLV-treated mice (n = 7) and some of the ICLV-treated ones (n = 3) were subjected to 70% partial hepatectomy. We validated the concept that partial hepatectomy resulted in an increased incorporation of bromodeoxyuridine consistent with *de novo* induction of hepatocyte proliferation (data not shown). In the recovering mice, FIX levels remained comparable to those measured before hepatectomy in the ICLV-treated group (Fig. 3A). In contrast, although FIX levels in the IDLV-treated mice were relatively stable before partial hepatectomy, they significantly declined shortly afterwards in the interval between weeks 12 and 20 (*P* < 0.05; Fig. 3B). Instead, there was no statistically significant decline in FIX expression in another IDLV-treated cohort that was not subjected to PHX in the same time interval (weeks 12-20). Nevertheless, FIX expression had become subtherapeutic after 1 year (Fig. 3C).

Overall, these data indicate that IDLVs, though less efficient than their ICLV counterparts at expressing transgenes from human and murine hepatocytes, can support therapeutically relevant levels of transgene expression *in vivo*. This finding warranted a further assessment of the risk/benefit ratio of the IDLV platform. The posthepatectomy drop in FIX levels observed in IDLV-treated mice suggested that FIX expression could be attributed mainly to nonintegrated vector episomes. Thus, we investigated the residual IDLV integration frequency.

### IDLVs Integrate Only to Background Levels and With Features Incompatible With Residual Catalytic Activity of the HIV Integrase

**Fig. 4:**
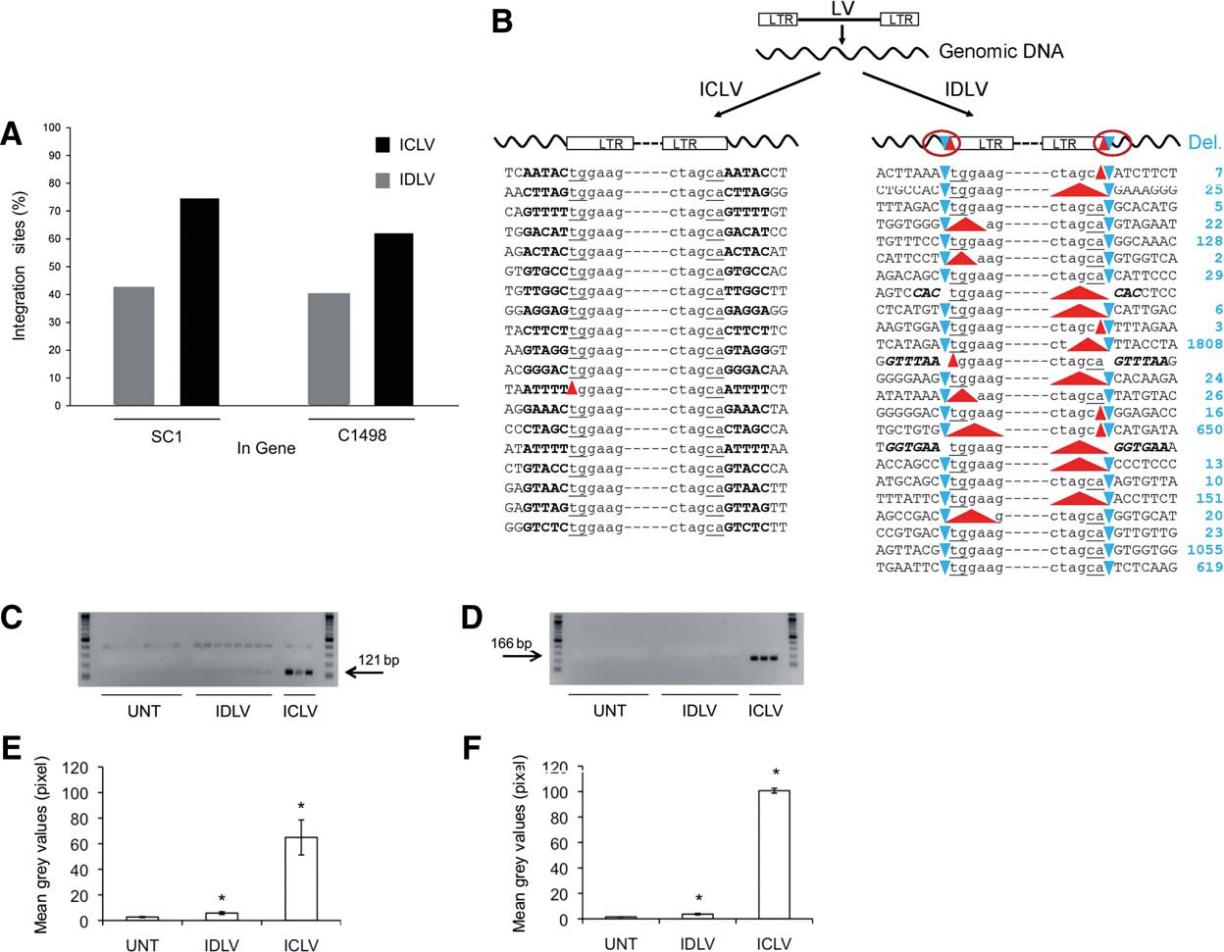
Assessment of residual IDLV integration *in vitro* and in the liver (see also Supporting Figure 2-4 and Table 1) (A) Distribution of integration sites determined by LAM-PCR that were located in gene coding regions of ICLV- (black bars) or IDLV- (grey bars) transduced mouse SC-1 and C1498 cells (SC-1 cells: 533 IDLV IS, 2419 ICLV IS; C1498: 271 IDLV IS, 898 ICLV IS). (B) LTR deletions (red triangles) and genomic deletions (blue triangles) identified in IDLV and ICLV integrants from which both the 5' and 3' genomic flanking region were identified by LAM-PCR. The terminal CA dinucleotide (underlined, GT at end of 5' strand LTR, complementary to CA at end of 3' strand LTR) is shown if exist. The 5 bp direct repeat of host DNA flanking the provirus is delineated in bold. The number of deleted bp in host DNA is indicated (Del.). LTR: long terminal repeat. LV: lentiviral vector. B2-PCR product gel image for primer set 1 (C) and primer set 2 (D). The arrows indicate the position of the respective PCR bands; densitometric analysis of the B2-PCR bands received with primer set 1 (E) and primer set 2 (F) in the untreated (UNT), IDLV- and ICLV-treated liver samples. Statistically significant differences (*) were found between ICLV and IDLV (t-test: *P* < 10^-4^ and *P* < 10^-11^) as well as between UNT and IDLV (t-test: *P* < 0.002) with both primer sets 1 and 2, respectively. Abbreviation: UNT, untreated.

Because the D64V mutation reduces LV integration by 2 to 3 log (see also Fig. 1),^13^ low-level integration still occurs with this mutant. We therefore assessed residual IDLV integration *in vitro* and in the treated livers. First, two murine cell lines were transduced with IDLV.GFP and were subsequently cultured for 8 weeks to dilute out the episomal forms. The GFP^+^ cells were then selectively enriched by fluorescence-activated cell sorting (FACS). We then applied nonrestrictive and standard, 5′- and 3′-LTR–mediated LAM-PCR strategies^21^,^22^ along with 454 pyrosequencing to analyze IDLV integration in the bulk positive cell populations and in single cell–derived clones. A comprehensive, large-scale analysis of more than 800 unique, mappable IDLV ISs on the mouse genome revealed close to random genomic integration of IDLVs without any preference for gene coding regions (Fig. 4A). In contrast, the 3317 unique ICLV ISs that could be mapped to the genome showed the characteristic lentiviral IS profile with gene coding regions as preferred targets. To distinguish noncanonical integration from residual integrase activity–mediated integration, we screened our IDLV and ICLV integration data for the presence of the characteristic 5-bp direct repeat of host DNA flanking the proviral terminal CA dinucleotide as a hallmark of integrase activity. For IDLVs, we retrieved the 5′- and 3′-vector–host genome junctions in 22 instances from the bulk cell populations (statistical considerations make it extremely unlikely that such junctions could come from two distinct integration events) and in 2 instances from single cell–derived clones. Seventeen of the 24 integrants showed loss of the CA nucleotide at least at one end of the vector. All 24 integrants revealed a partial deletion of LTR and/or genomic sequences, and no 5-bp direct repeat could be detected in any of these vector-genome junctions (Fig. 4B). In contrast, 18 of 19 ICLV integrations for which both sequence ends were mapped revealed neither CA dinucleotide deletions nor LTR sequence deletions, and they harbored the typical 5-bp direct repeat without genomic deletions. In agreement with these data, screening for LTR sequence deletions in our complete IS data sets showed that one-third of all IDLV integrations had a partial loss of the LTR sequence, whereas LTR deletions accounted for only approximately 2% of all ICLV sequences. Interestingly, our LAM-PCR screening for potentially broken linear and circular vector forms, which used vector internal primers binding to the GFP transgene or vector backbone, did not show any sign of integration (data not shown). Our data provide direct molecular evidence that the background integration of D64V IDLVs is not mediated by residual catalytic activity of the mutant integrase.

To determine the residual IDLV integration frequency in the treated livers, we first performed a semiquantitative nested PCR to amplify host DNA–vector junctions with short interspersed (SINE) nuclear B2 repeat sequences that are scattered throughout the mouse genome (Supporting Information Fig. 2). Although the livers from ICLV-treated mice yielded intense bands for the 5′- and 3′-vector–host genome junctions, the livers from IDLV-treated mice showed bands of much lower intensity consistent with low-level residual integration (Fig. 4C-F). We further retrieved vector sequences from the treated livers at 6 to 12 weeks post-injection by highly sensitive LAM-PCR and deep sequencing. The number of unique ISs found in the IDLV-treated liver samples was significantly lower than the number in the ICLV samples. In the IDLV liver samples (n = 16), a total of 35 unique, mappable ISs could be recovered versus 785 ISs in the ICLV samples (n = 4, *P* < 10^−9^) with an almost 100-fold lower frequency of retrieval (Table 1). Accordingly, we retrieved an excess of 2-LTR junctions from IDLV-treated liver samples versus ICLV-treated ones, and this was suggestive of the presence of episomal vector forms (2-LTR junctions: 54.5% in IDLV samples and 2.8% in ICLV samples; Table 1 and Supporting Information Fig. 4). Consistent with the *in vitro* IS data, the few retrieved integrations did not show enrichment for genes and frequently showed deletion in the LTR ends (25.7% in IDLV samples and 0.6% in ICLV samples; Table 1 and Supporting Information Fig. 3).

**Table 1:** Deep Sequencing of ICLV- and IDLV-Transduced Liver Samples

Vector	Raw Sequence Reads	1-LTR Amplicon/Internal Control	2-LTR Amplicon	Reads With an LTR^+^, ≥20-nt Genomic Sequence	Unique Mappable ISs	LTR Deleted at the Genome Junction by >3 nt
ICLV	**2787**	**211**	**51**	**1688**	**152**	**2 (10, 11 nt)**
	2179	13	0	244	244	1 (22 nt)
	3169	896	53	101	101	
	2822	250	258	288	288	2 (8, 23 nt)
IDLV	**1451**	**284**	**760**	**340**	**0**	
	**971**	**530**	**237**	**186**	**2**	
	**1227**	**373**	**82**	**767**	**0**	
	**1365**	**466**	**154**	**26**	**2**	**1 (21 nt)**
	**2264**	**371**	**838**	**841**	**2**	
	**2752**	**400**	**996**	**1322**	**1**	
	**1349**	**221**	**795**	**308**	**4**	
	**1173**	**193**	**587**	**277**	**3**	**1 (11 nt)**
	**2809**	**347**	**1538**	**502**	**1**	**1 (8 nt)**
	**1722**	**215**	**801**	**421**	**4**	**2 (19, 24 nt)**
	3494	350	2124	916	4	2 (14, 21 nt)
	3260	223	2397	500	2	
	3291	316	1799	621	4	1 (25 nt)
	2771	330	1963	232	2	
	2882	275	2795	318	0	
	3251	241	1787	808	4	1 (8 nt)

This table shows 454 sequencing results for ICLV- and IDLV-transduced liver samples. GFP-transduced samples are indicated in bold, and FIX-transduced samples are indicated in regular type. The 1-LTR amplicon and vector internal control fragments (resulting from the 3′-LTR-U3 LAM-PCR primer annealing at 5′-LTR-U3) could not be distinguished by sequencing. Notably, the 2-LTR amplicon LAM-PCR product (restriction enzyme Tsp509I, AATT) exhibited a length of only 18 bp without an LTR and linker sequence, and this resulted in the overrepresentation of sequenced 2-LTR amplicons versus the 1-LTR amplicon and the internal control (Supporting Information Fig. 4).

Overall, these studies indicate abrogation of the HIV integrase–dependent integration in IDLVs. The background integration in treated livers exhibits molecular features reminiscent of those described for plasmids and other types of episomal DNA. This genomic IS analysis further underscores the minimal risk of insertional mutagenesis by IDLVs.

### IDLV Delivery Can Tolerize the Recipient to Foreign Antigens

**Fig. 5:**
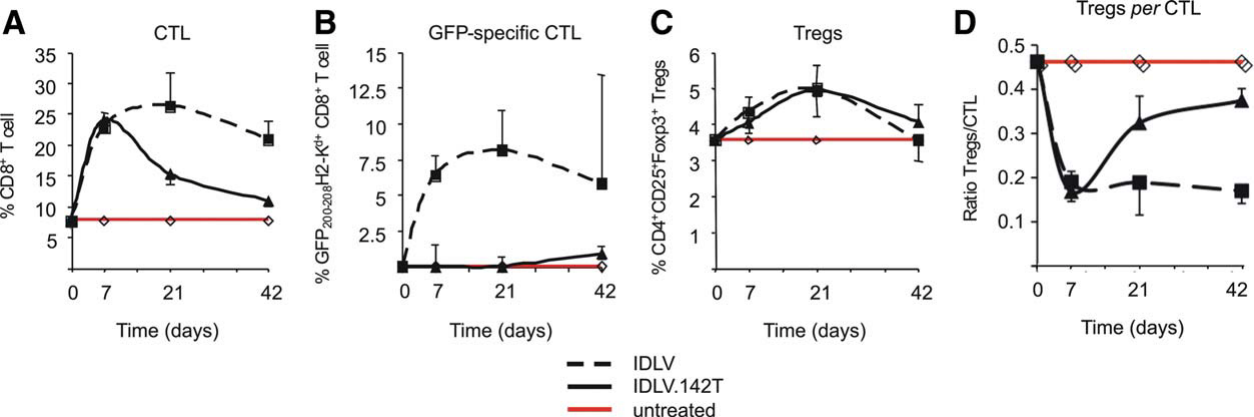
Kinetics of liver lymphocytes infiltrate after IDLV gene transfer Mononuclear cells (MNC) were isolated from the liver of mice injected with matched doses of hepatocyte targeted ET.GFP.142T IDLV (IDLV.142T) or control PGK.GFP IDLV (IDLV) at the indicated time post-injection (IDLV.142T n = 20, IDLV n = 19, untreated n = 9 in 3 indipendent experiment) (A) Frequency of CD8^+^ T, (B) GFP_200-208_-pentamer-positive, and (C) CD4^+^CD25^+^Foxp3^+^ Tregs infiltrating cells was evaluated by flow cytometry. Data are expressed as the mean % ± SD (n = 3 per group per time point; a representative experiment out of 3 is shown). (D) Ratio of T regs to CD8+ T cell. Abbreviation: CTL, cytotoxic T lymphocyte.

We then investigated whether hepatocyte-targeted expression by IDLVs induces transgene-specific immunological tolerance. We monitored liver infiltration by lymphocyte subpopulations after the injection of IDLV.ET.GFP.142T or a control IDLV expressing GFP from a constitutive PGK promoter, which was expected to induce a GFP-specific immune response^6^ (n = 20 for the IDLV.142T group, n = 19 for the IDLV group, and n = 9 for the untreated group). CD8^+^ T cell infiltration was well detected 1 week after IDLV treatment independently of the presence or absence of miR-142 regulation. However, although the CD8^+^ T cells persisted at a high frequency in the livers of mice treated with the unregulated vector, they decreased substantially 3 weeks post-injection in IDLV.142T-treated mice (Fig. 5A). These findings were paralleled by the induction of GFP-specific CD8^+^ T cells (Fig. 5B). The frequency of CD4^+^CD25^+^FOXP3^+^ regulatory T cells (Tregs; FOXP3 = forkhead box P3) increased in the liver after treatment with both IDLV types 3 weeks after vector administration; however, the ratio of Tregs to CD8^+^ effector cells was much higher in the mice treated with miR-142-regulated vector than in control vector treated mice (Fig. 5C,D).

**Fig. 6:**
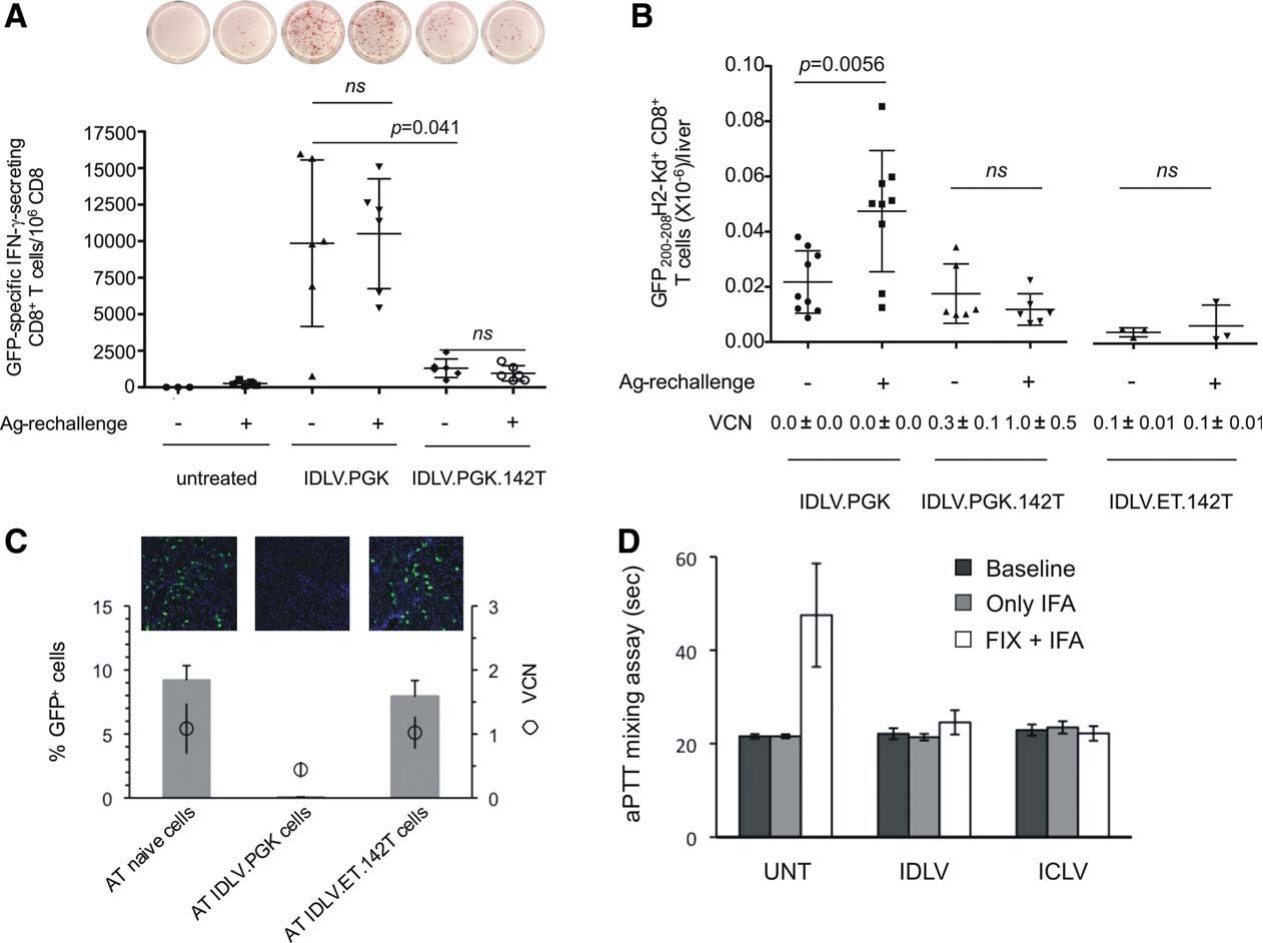
Transgene-specific tolerance after IDLV liver gene transfer (A) Secondary immune response to the vector-encoded antigen was assessed by frequency of IFN-γ-producing, GFP-specific CD8^+^ T cells in the spleen of mice subjected to antigen re-challenge (by intramuscular vaccination with GFP-encoding plasmids) 6 weeks after IDLV treatment. Single values are plotted and the mean ± SD number of GFP-specific CD8^+^ T cells *per* 10^6^ total CD8^+^ T cells is shown (n = 6 per group). Representative wells are shown on top. (B) Quantification of the CD8^+^ GFP_200-208_-pentamer-positive T cells infiltrating the liver of mice treated with the indicated IDLV (IDLV.PGK n = 9; IDLV.PGK.142T n = 6; IDLV.ET.142T n = 3) after antigen re-challenge. Single values are plotted and mean ± SD is shown. Mean ± SD VCN in the liver is indicated. (C) Morphometric analysis of GFP^+^ hepatocytes and VCN in the liver of immune-deficient mice previously injected with ET.GFP.142T ICLV and adoptively transferred (AT) with cells derived from naïve, IDLV.PGK- and IDLV.ET.142T-treated mice (from 5A-C). Mean ± SEM of n = 3 mice (left axis). Circles show mean ± SEM VCN (right axis). A representative field is shown on top. Note that residual vector DNA in the liver of mice in which GFP-expressing hepatocytes were completely cleared can be ascribed to the persistence of vector genomes in transduced macrophages and endothelial cells that did not express GFP. (D) Plasma samples from the various groups of animals were screened for anti-FIX neutralizing antibodies using an aPTT mixing assay using a positive control of 2.6 Bethesda Units (BU) and a known negative control. Samples were assayed in a blinded fashion. Groups include FIX KO mice treated with the indicated ET.FIX.142T vector or untreated (UNT) 12 weeks after vector administration, with or without challenge with canine FIX and IFA. Only untreated mice receiving FIX + IFA had detectible neutralizing anti-FIX antibodies and all were greater than 2.6 BU. Abbreviations: Ag, antigen; ns, not significant; UNT, untreated.

To investigate whether active tolerance was established, we challenged the mice by intramuscular vaccination with GFP-encoding plasmids and evaluated the frequency of GFP-specific CD8^+^ T cells in the spleen and liver (Fig. 6A,B). Although the low frequency of GFP-specific CD8^+^ T cells induced in the spleens and livers of IDLV.142T-treated mice did not increase upon rechallenge, there were many more antigen-specific effectors in the control, unregulated IDLV-treated mice after the primary (IDLV) and secondary challenges (intramuscular plasmid). In addition, residual levels of vector genomes and GFP expression were still detectable in the livers of mice previously treated with IDLV.142T after rechallenge (Fig. 6B and data not shown).

To further confirm that IDLV.142T is able to direct the immune system toward the induction of immune tolerance to transgene antigens, we reconstituted immune-deficient recipients through the adoptive transfer of immune cells derived from either immunized (IDLV-treated) or tolerized (IDLV.142T-treated) mice (Fig. 6C). Rag2^−/−^γ-chain^−/−^ mice (Rag2 = recombination activating gene 2; γ-chain = cytokine receptor common subunit gamma) were, therefore, first transduced with hepatocyte-targeted ICLVs (ET.GFP.142T) to obtain robust GFP expression in hepatocytes. A pool of splenocytes and liver lymphocytes derived from naive, control IDLV-treated or IDLV.142T-treated animals was transferred 1 month later. Although comparable amounts of GFP-expressing hepatocytes were detected in the mice reconstituted with naive cells or cells derived from tolerant (IDLV.142T-treated) mice, a complete clearance of GFP^+^ hepatocytes was observed in recipient mice reconstituted with cells derived from control, unregulated IDLV-treated mice, and this was accompanied by a loss of vector DNA. These results indicate that only those cells derived from control mice exhibited the full effector potential upon adoptive transfer, whereas the effector cells derived from tolerized mice (IDLV.142T-treated) were kept in check by the induced regulatory compartment. Taken together, these data indicate that GFP-specific immunological tolerance was established.

To assess whether immune tolerance could be induced by IDLVs toward a therapeutically relevant secreted antigen, we evaluated the anti-FIX immune response in hemophilic mice treated with FIX-encoding IDLV.142T. Activated partial thromboplastin time (aPTT)–based Bethesda assays showed no anti-FIX inhibitory activity (Fig. 6D). Importantly, when these IDLV.142T-treated mice were challenged with FIX protein in incomplete Freund adjuvant (IFA), they remained negative for anti-FIX neutralizing antibodies. In contrast, hemophilic mice that were not injected with any vector but were immunized with FIX and IFA showed the induction of neutralizing antibodies (the Bethesda titer was >2.6 Bethesda units in these mice, whereas it was below the level of detection in all recipient mice of the other cohorts). These results indicate that FIX-specific immunological tolerance was established.

### IDLV Delivery Induces Antigen-Specific Tregs

**Fig. 7:**
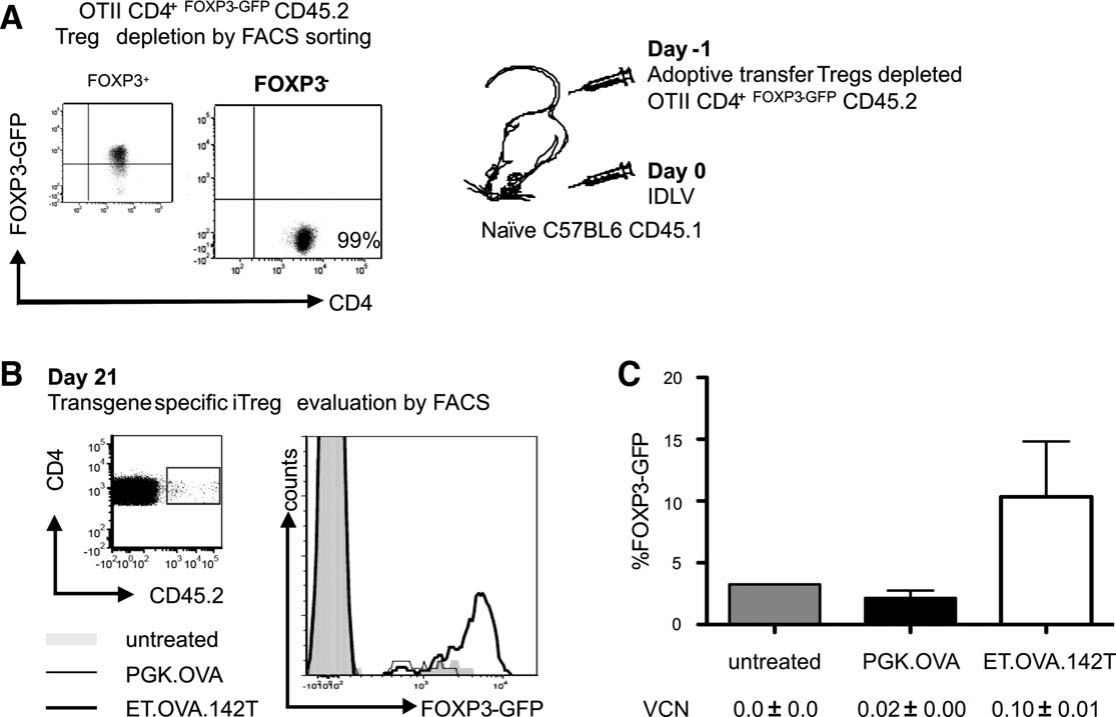
Induction of transgene-specific Tregs by IDLV treatment CD4^+^ cells isolated from OTII Ly5.2 ^Foxp3-GFP^ transgenic mice were FACS-sorted to remove GFP^+^ cells obtaining an homogeneous population of CD4^+^ non-regulatory T cells with a unique antigen specificity (OVA_323-339_ presented in IA^b^ molecule). (A) Tregs-depleted OTII CD4^+^GFP^-^ (2.5&times;10^6^/mouse) were adoptively transfer intravenously into naïve C57BL/6 Ly5.1 recipient mice one day before the injection of IDLV.PGK (n = 3) or IDLV.ET.142T (n = 3) encoding for OVA. Three weeks after IDLV administration livers were harvested and infiltrating lymphocytes isolated. OVA-specific induced Tregs (iTregs) were measured as GFP^+^ cells gated on CD4^+^Ly5.2^+^. (B) a representative histogram and (C) mean % induced Tregs ± SEM is reported.

To determine whether the antigen specificity of liver-infiltrating Tregs is affected by IDLV.142T administration, the antigen-driven conversion of naive CD4^+^FOXP3^−^ T cells into FOXP3^+^ Tregs was evaluated. To this end, we adoptively transferred naive CD4^+^ T cells obtained from double-transgenic mice for an OVA-specific T cell receptor (major histocompatibility complex II–restricted, OTII) and a GFP reporter knock-in downstream of the *Foxp3* promoter. OTII CD4^+ FOXP3-GFP^ Ly5.2 T cells were isolated, FACS-sorted to deplete FOXP3^+^ cells, and adoptively transferred into naive Ly5.1 recipient mice before the injection of OVA-encoding IDLVs or IDLV.142T (Fig. 7A). Three weeks later, the recipient mice were euthanized, and GFP expression, used as a surrogate marker for FOXP3, was determined in OTII Ly5.2 CD4^+^ T cells. A significantly higher frequency of OVA-specific Tregs was detected specifically in the IDLV.142T-treated mice versus the untreated or control, unregulated IDLV-treated mice (Fig. 7B). These results indicate that the pattern of OVA expression driven by miR-142–regulated IDLVs in the liver favored the conversion of transgene-specific naive CD4^+^ T cells into transgene-specific FOXP3^+^-induced Tregs (Fig. 7B,C).

Overall, these data indicate that hepatocyte-targeted expression of foreign antigens, including intracellular or secreted, therapeutically relevant (FIX) and model antigens (GFP and OVA), by IDLVs can result in a state of transgene-specific immunological tolerance due to a strong contraction of the transgene-specific effector compartment and the induction of the transgene-specific regulatory compartment.

## Discussion

IDLVs are emerging as an attractive platform for transgene expression for several purposes.^23^ This platform harnesses the pantropism and proficiency of LV transduction without relying on integration and permanent modification of the cellular genome. Here we show that IDLVs can be used to express transgenes for a window of time in the liver as long as the expression is stringently targeted to hepatocytes with transcriptional and miR-142–mediated regulation. Although the IDLV expression efficiency is lower than that observed for ICLVs, the expression levels are sufficient to induce immune tolerance and achieve prolonged therapeutic effects in a clinically relevant disease model. Moreover, the nonintegrating feature of the platform provides important safety advantages because of the low risk of genotoxicity and the reversibility of transgene expression.

The declining transgene expression of IDLVs in proliferating cells has been mostly ascribed to the progressive loss of episomal DNA from the cell nucleus during mitosis versus the stably integrated provirus of ICLVs. Here, we have reliably quantified the contents of reverse-transcribed vector genomes and the transgene expression level in transduced dividing or quiescent cells, and we have shown that the expression efficiency per genome copy is significantly lower for IDLVs versus ICLVs in hepatocytes. This may be due to diffusion of the episomes to nuclear areas that are not involved in active transcription, inefficient chromatin deposition, or enrichment with histone modifications typical of transcriptionally silenced chromatin.^24^ This is in contrast to what has been reported in other quiescent tissues, such as the retina and the central nervous system, and may indicate tissue-specific factors affecting the expression and stability of episomal IDLVs. It is conceivable that the incorporation of additional genomic elements into the IDLV backbone may improve the expression proficiency and nuclear stability.

We have shown that IDLV background integration occurs by mechanisms incompatible with residual activity of the mutant integrase because they often present deletions in the LTR ends and lack the typical flanking genomic repeats at the insertion site. These events may be mediated by nonhomologous end joining of linear episomes to sites of chromosomal breakage.^25^ Although we cannot rule out a contribution to the observed sustained FIX expression by these integrated IDLVs, the significant decline of FIX expression with the induction of hepatocyte proliferation after partial hepatectomy strongly suggests that *in vivo* expression is mostly mediated by the episomal forms.

The significantly lower expression of IDLVs versus ICLVs represents a limiting factor for their application to stable therapeutic gene replacement in the liver, at least in the current design. However, IDLVs may be considered whenever reversible gene transfer is preferable (e.g., the testing of a new gene therapy approach), especially if the clinical setting imposes high safety bars in the face of existing treatment options (e.g., hemophilia) or the biological effects of gene-based delivery are difficult to predict or may entail substantial toxicity. Here we demonstrate the therapeutic potential of inducing a prolonged window of FIX expression in the plasma of hemophilic mice. Apparently, FIX expression was more prolonged than GFP expression after hepatic transduction with IDLVs. This possibly reflects the higher vector doses and/or differences in the detection limits of the assays used to quantify the expression of the respective transgene products. IDLVs may also be used for hepatic expression of therapeutic proteins, such as interferon (IFN) and other cytokines, in chronic viral hepatitis or hepatic tumors; gene-based delivery may provide therapeutic concentrations of the factor at the disease site with limited systemic exposure and only for a defined window of time.^26^,^27^

Hepatic gene transfer has been associated with the induction of immunological tolerance to the transgene product with several vector platforms.^28-30^ In contrast to other viral vectors used in gene therapy, most subjects are immunologically naive to the IDLV vector components, so it is unlikely that IDLV-transduced hepatocytes would be recognized by vector-specific cytotoxic T cells. Here we demonstrate a major accompanying benefit of hepatocyte-targeted IDLV gene transfer: the induction of transgene-specific Tregs and active tolerance to the transgene product. Most importantly, this response may extend beyond the duration of vector-mediated transgene expression and improve the efficacy of existing protein replacement therapy by preventing the induction of neutralizing antibodies, which represent one of the major hurdles of this treatment. Indeed, the *in vivo* induction of antigen-specific Tregs by microRNA-regulated IDLVs may well represent their most attractive feature to date, and it provides an intriguing contrast to the immunogenic nature of unregulated IDLV delivery, which is currently being explored for the design of improved virus-based vaccines.^31^,^32^ Although IDLVs are less efficient at expressing the transgene in hepatocytes in comparison with their integrating counterparts, they are equally efficient at inducing transgene-specific tolerance, and this suggests that the pattern of transgene expression (and not the level) plays a crucial role in directing the immune system response in this setting.

A broad application of hepatocyte-targeted expression by ICLVs for immune modulation is currently limited by the concerns associated with integration in the target cell genome. IDLVs are advantageous for this purpose and could be exploited in inverse vaccination strategies to tolerize individuals to protein replacement or gene therapies and prevent the development of autoimmune disease in at-risk individuals.^33^ The present study sets the stage for further testing in preclinical models.

## Abbreviations

Ag: antigen
aPTT: activated partial thromboplastin time
CTL: cytotoxic T lymphocyte
ET: enhanced transthyretin
FACS: fluorescence-activated cell sorting
FIX: coagulation factor IX
FOXP3: forkhead box P3
γ-chain: cytokine receptor common subunit gamma
Gag: group-specific antigen
GFP: green fluorescent protein
HIV: human immunodeficiency virus
ICLV: integrase-competent lentiviral vector
IDLV: integrase-defective lentiviral vector
IFA: incomplete Freund adjuvant
IFN: interferon
IS: integration site
LAM-PCR: linear amplification–mediated polymerase chain reaction
LTR: long terminal repeat
LV: lentiviral vector
MFI: mean fluorescence intensity
miR-142: microRNA 142
MOI: multiplicity of infection
ns: not significant
OVA: ovalbumin
PCR: polymerase chain reaction
PGK: phosphoglycerokinase
PHX: partial hepatectomy
Rag2: recombination activating gene 2
Treg: regulatory T cell
UNT: untreated
UTR: untranslated region
VCN: vector copy number

## References

[1] Nguyen TH, Ferry N (2004). Liver gene therapy: advances and hurdles. Gene Ther.

[2] Manno CS, Pierce GF, Arruda VR, Glader B, Ragni M, Rasko JJ (2006). Successful transduction of liver in hemophilia by AAV-factor IX and limitations imposed by the host immune response. Nat Med.

[3] Pierce GF, Lillicrap D, Pipe SW, Vandendriessche T (2007). Gene therapy, bioengineered clotting factors and novel technologies for hemophilia treatment. J Thromb Haemost.

[4] Wasserfall CH, Herzog RW (2009). Gene therapy approaches to induce tolerance in autoimmunity by reshaping the immune system. Curr Opin Investig Drugs.

[5] Luth S, Huber S, Schramm C, Buch T, Zander S, Stadelmann C (2008). Ectopic expression of neural autoantigen in mouse liver suppresses experimental autoimmune neuroinflammation by inducing antigen-specific Tregs. J Clin Invest.

[6] Annoni A, Brown BD, Cantore A, Sergi LS, Naldini L, Roncarolo MG (2009). *In vivo* delivery of a microRNA-regulated transgene induces antigen-specific regulatory T cells and promotes immunologic tolerance. Blood.

[7] Brown BD, Venneri MA, Zingale A, Sergi Sergi L, Naldini L (2006). Endogenous microRNA regulation suppresses transgene expression in hematopoietic lineages and enables stable gene transfer. Nat Med.

[8] Brown BD, Cantore A, Annoni A, Sergi LS, Lombardo A, Della Valle P (2007). A microRNA-regulated lentiviral vector mediates stable correction of hemophilia B mice. Blood.

[9] VandenDriessche T, Thorrez L, Naldini L, Follenzi A, Moons L, Berneman Z (2002). Lentiviral vectors containing the human immunodeficiency virus type-1 central polypurine tract can efficiently transduce nondividing hepatocytes and antigen-presenting cells *in vivo*. Blood.

[10] Montini E, Cesana D, Schmidt M, Sanvito F, Bartholomae CC, Ranzani M (2009). The genotoxic potential of retroviral vectors is strongly modulated by vector design and integration site selection in a mouse model of HSC gene therapy. J Clin Invest.

[11] Cartier N, Hacein-Bey-Abina S, Bartholomae CC, Veres G, Schmidt M, Kutschera I (2009). Hematopoietic stem cell gene therapy with a lentiviral vector in X-linked adrenoleukodystrophy. Science.

[12] Naldini L, Blomer U, Gallay P, Ory D, Mulligan R, Gage FH (1996). *In vivo* gene delivery and stable transduction of nondividing cells by a lentiviral vector. Science.

[13] Leavitt AD, Robles G, Alesandro N, Varmus HE (1996). Human immunodeficiency virus type 1 integrase mutants retain *in vitro* integrase activity yet fail to integrate viral DNA efficiently during infection. J Virol.

[14] Nightingale SJ, Hollis RP, Pepper KA, Petersen D, Yu XJ, Yang C (2006). Transient gene expression by nonintegrating lentiviral vectors. Mol Ther.

[15] Vargas J, Gusella GL, Najfeld V, Klotman ME, Cara A (2004). Novel integrase-defective lentiviral episomal vectors for gene transfer. Hum Gene Ther.

[16] Saenz DT, Loewen N, Peretz M, Whitwam T, Barraza R, Howell KG (2004). Unintegrated lentivirus DNA persistence and accessibility to expression in nondividing cells: analysis with class I integrase mutants. J Virol.

[17] Yanez-Munoz RJ, Balaggan KS, MacNeil A, Howe SJ, Schmidt M, Smith AJ (2006). Effective gene therapy with nonintegrating lentiviral vectors. Nat Med.

[18] Philippe S, Sarkis C, Barkats M, Mammeri H, Ladroue C, Petit C (2006). Lentiviral vectors with a defective integrase allow efficient and sustained transgene expression *in vitro* and *in vivo*. Proc Natl Acad Sci U S A.

[19] Lombardo A, Genovese P, Beausejour CM, Colleoni S, Lee YL, Kim KA (2007). Gene editing in human stem cells using zinc finger nucleases and integrase-defective lentiviral vector delivery. Nat Biotechnol.

[20] Apolonia L, Waddington SN, Fernandes C, Ward NJ, Bouma G, Blundell MP (2007). Stable gene transfer to muscle using non-integrating lentiviral vectors. Mol Ther.

[21] Gabriel R, Eckenberg R, Paruzynski A, Bartholomae CC, Nowrouzi A, Arens A (2009). Comprehensive genomic access to vector integration in clinical gene therapy. Nat Med.

[22] Schmidt M, Schwarzwaelder K, Bartholomae C, Zaoui K, Ball C, Pilz I (2007). High-resolution insertion-site analysis by linear amplification-mediated PCR (LAM-PCR). Nat Methods.

[23] Banasik MB, McCray PB (2010). Integrase-defective lentiviral vectors: progress and applications. Gene Ther.

[24] Kantor B, Ma H, Webster-Cyriaque J, Monahan PE, Kafri T (2009). Epigenetic activation of unintegrated HIV-1 genomes by gut-associated short chain fatty acids and its implications for HIV infection. Proc Natl Acad Sci U S A.

[25] Miller DG, Petek LM, Russell DW (2004). Adeno-associated virus vectors integrate at chromosome breakage sites. Nat Genet.

[26] De Palma M, Mazzieri R, Politi LS, Pucci F, Zonari E, Sitia G (2008). Tumor-targeted interferon-alpha delivery by Tie2-expressing monocytes inhibits tumor growth and metastasis. Cancer Cell.

[27] Berraondo P, Ochoa L, Crettaz J, Rotellar F, Vales A, Martinez-Anso E (2005). IFN-alpha gene therapy for woodchuck hepatitis with adeno-associated virus: differences in duration of gene expression and antiviral activity using intraportal or intramuscular routes. Mol Ther.

[28] LoDuca PA, Hoffman BE, Herzog RW (2009). Hepatic gene transfer as a means of tolerance induction to transgene products. Curr Gene Ther.

[29] Racanelli V, Rehermann B (2006). The liver as an immunological organ. HEPATOLOGY.

[30] Mingozzi F, Liu YL, Dobrzynski E, Kaufhold A, Liu JH, Wang Y (2003). Induction of immune tolerance to coagulation factor IX antigen by *in vivo* hepatic gene transfer. J Clin Invest.

[31] Negri DR, Michelini Z, Baroncelli S, Spada M, Vendetti S, Buffa V (2007). Successful immunization with a single injection of non-integrating lentiviral vector. Mol Ther.

[32] Coutant F, Frenkiel MP, Despres P, Charneau P (2008). Protective antiviral immunity conferred by a nonintegrative lentiviral vector-based vaccine. PLoS One.

[33] Steinman L (2010). Inverse vaccination, the opposite of Jenner's concept, for therapy of autoimmunity. J Intern Med.

