# Supplementary figures and images for: Hepatocyte-Targeted Expression by Integrase-Defective Lentiviral Vectors Induces Antigen-Specific Tolerance in Mice with Low Genotoxic Risk
Source: Hepatology. 2011 May;53(5):1696–707. doi: 10.1002/hep.24230 (PMC3112259; doi:10.1002/hep.24230)

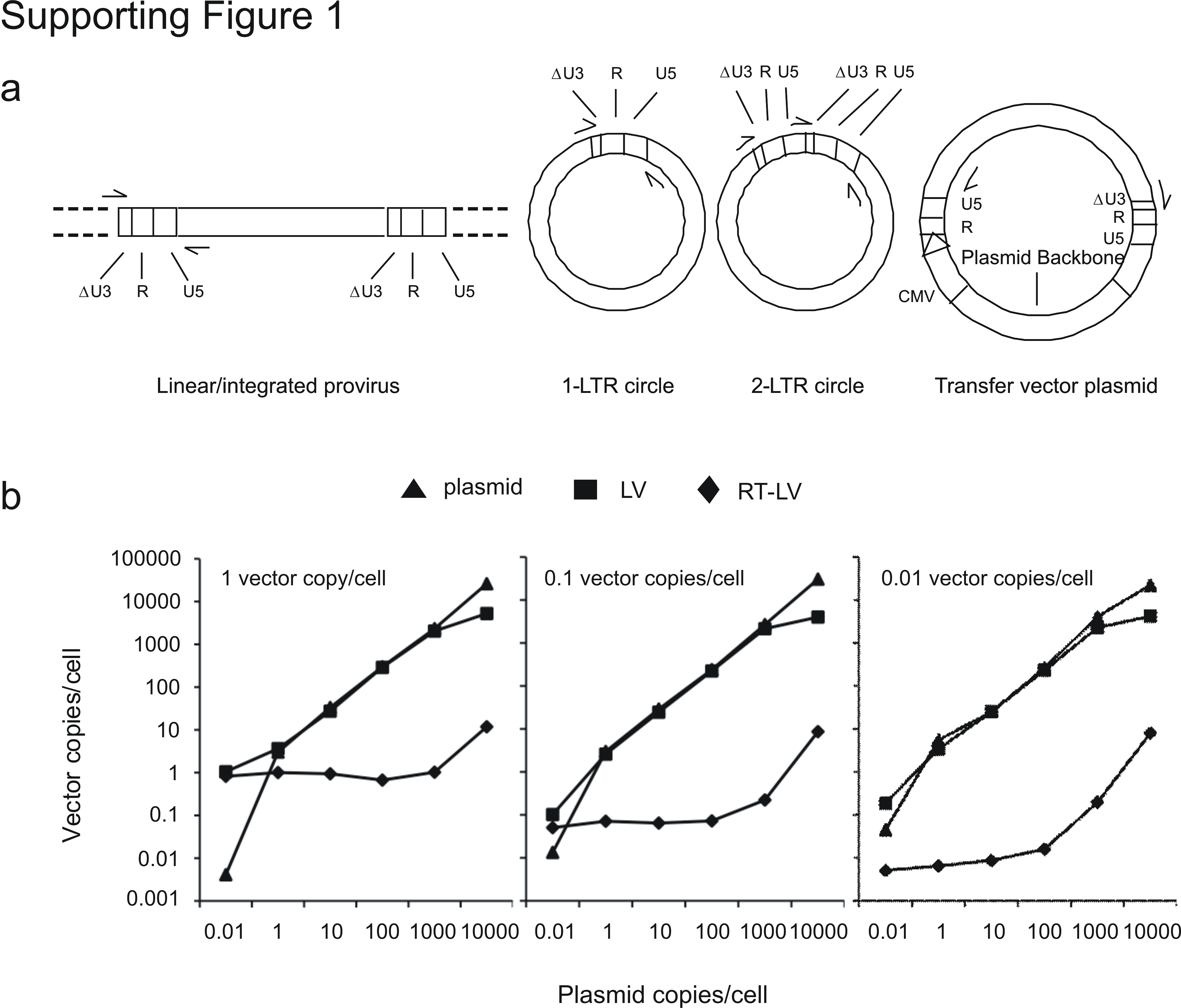

Supplement: Supplementary file 1 [file hep0053-1696-SD1.tif]

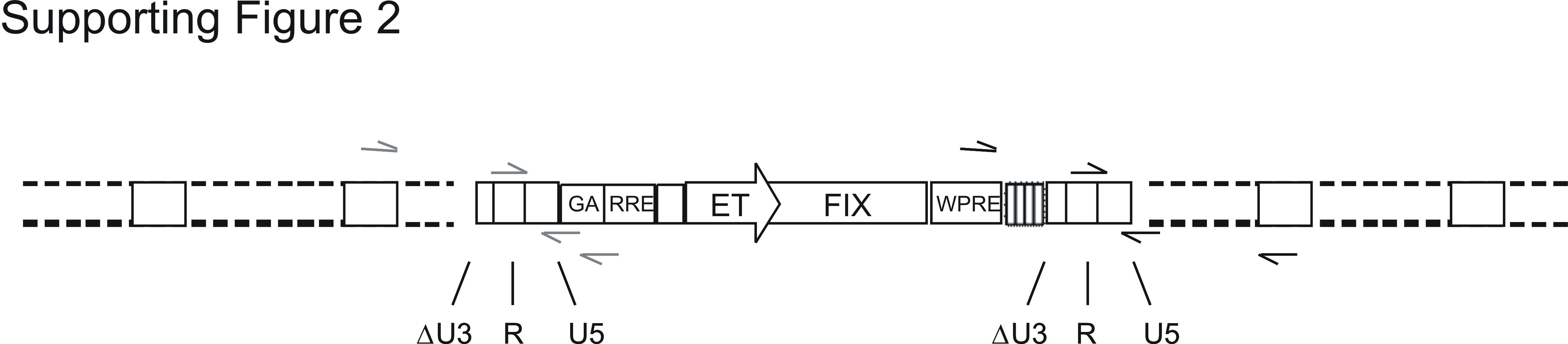

Supplement: Supplementary file 2 [file hep0053-1696-SD2.tif]

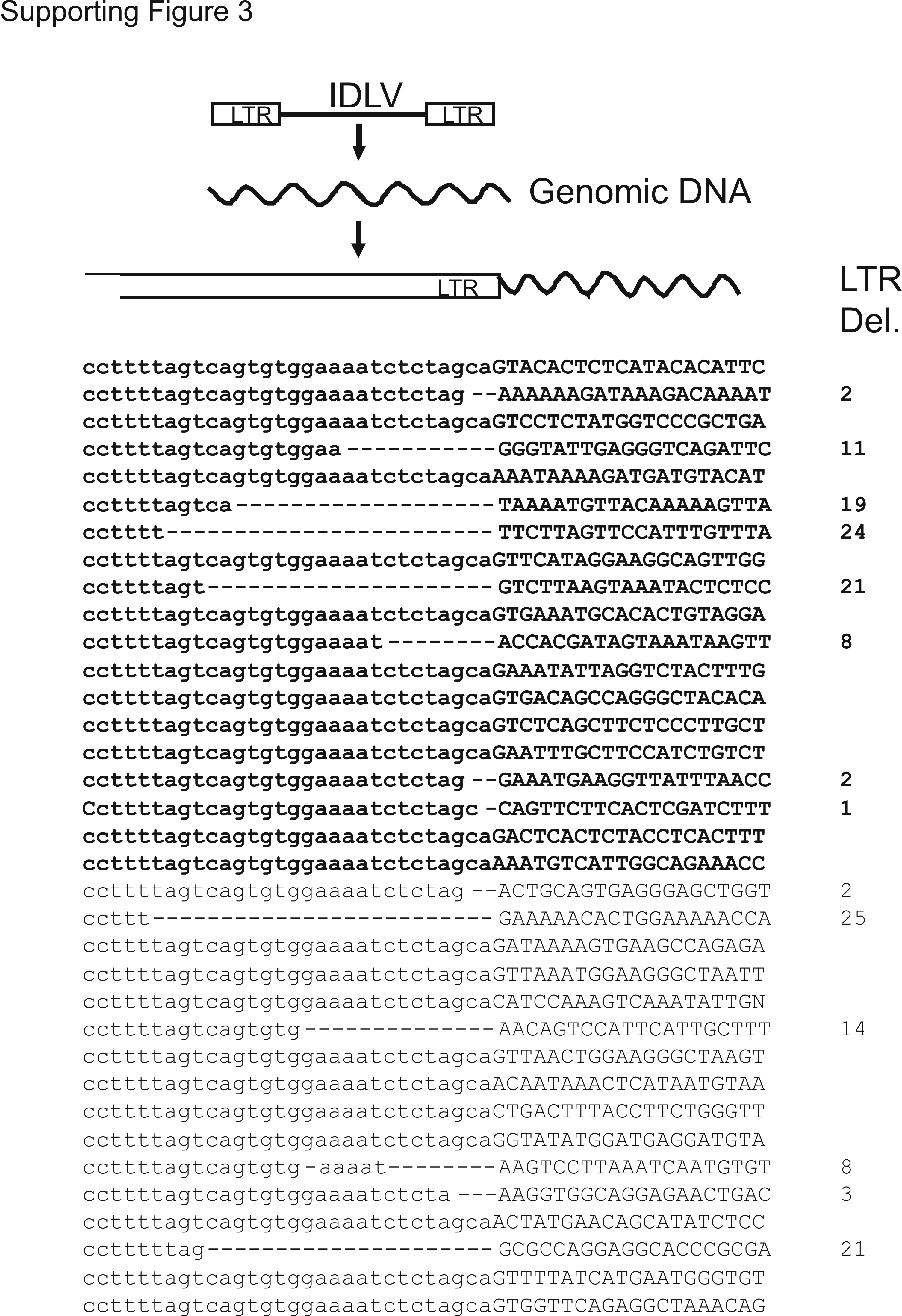

Supplement: Supplementary file 3 [file hep0053-1696-SD3.tif]

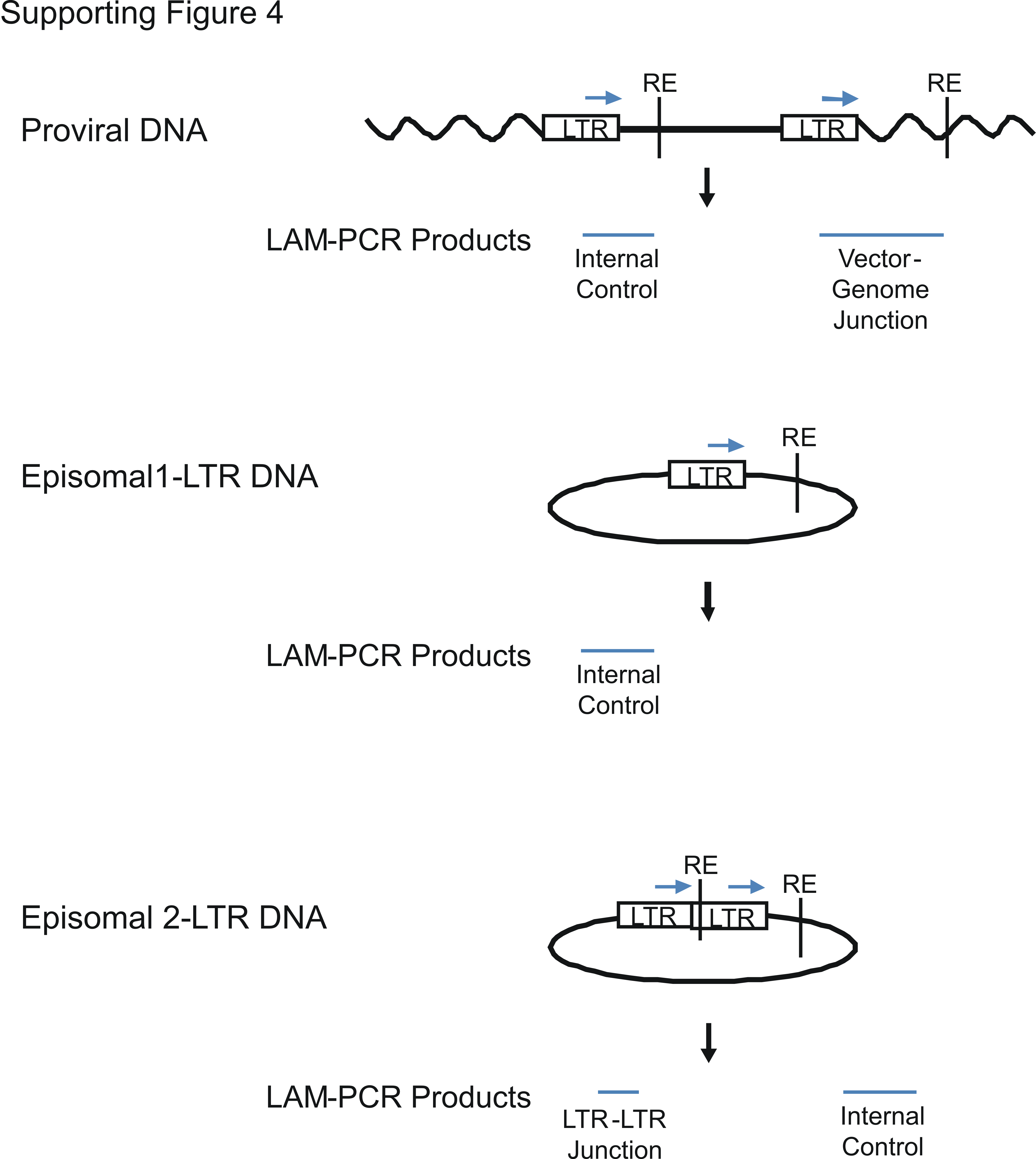

Supplement: Supplementary file 4 [file hep0053-1696-SD4.tif]
