# Supplementary material for: Hepatocyte-Targeted Expression by Integrase-Defective Lentiviral Vectors Induces Antigen-Specific Tolerance in Mice with Low Genotoxic Risk
Source: Hepatology. 2011 May;53(5):1696–707. doi: 10.1002/hep.24230 (PMC3112259; doi:10.1002/hep.24230)
Supplement: Supplementary file 5 [file hep0053-1696-SD5.doc]

**Supporting Table 1**

| **Vector** | **p24 (μg/ml)** | **Titer (TU/ml)** | **Infectivity (TU/ng p24)** |
| --- | --- | --- | --- |
| IDLV n=3 | 211±9 | 3.7E+09±1.2E+09 | 1.8E+04±3.6E+03 |
| ICLV n=3 | 162±28 | 5.0E+09±5.8E+08 | 3.9E+04±1.1E+04 |
| *p* | ns (0.1270) | ns (0.3606) | ns (0.0907) |

**Titer and infectivity of IDLV vs ICLV**. The table summarizes the p24, titer and infectivity of 3 different vector batches of ET.GFP.142T either packaged with an integrase-defective (IDLV) or integrase-competent (ICLV) configuration, as measured on 293T cells. Data are presented as mean ± standard error of the mean. ns: not significant (unpaired t test).
