# Supplementary material for: Hepatocyte-Targeted Expression by Integrase-Defective Lentiviral Vectors Induces Antigen-Specific Tolerance in Mice with Low Genotoxic Risk
Source: Hepatology. 2011 May;53(5):1696–707. doi: 10.1002/hep.24230 (PMC3112259; doi:10.1002/hep.24230)
Supplement: Supplementary file 6 [file hep0053-1696-SD6.doc]

**Supporting materials and methods**

**Vector production and titration**

At TIGET third-generation ICLV were produced by Ca_3_PO_4_ transfection into 293T cells, as previously described (1). Briefly, supernatants were collected, passed through a 0.22μm filter, and purified by ultracentrifugation. For IDLV production pMDLg/p.RRE.D64Vint was used instead of pMDLg/p.RRE as described (2). Vector concentration in reverse transcribed units (rTU) was determined on 293T cells 3 days after transduction, using an *ad hoc* qPCR, which selectively amplifies the reverse transcribed vector genome (both integrated and non-integrated) discriminating it from plasmid carried over from the transient transfection (RT-LV; ΔU3 sense: 5’-tcactcccaacgaagacaagatc-3’, gag antisense: 5’ gagtcctgcgtcgagagag-3’). The amount of human DNA loaded in the reaction was quantified with a qPCR designed to amplify the hTERT gene as described (2). Vector particles were measured by HIV-1 Gag p24 antigen immunocapture according to the manufacturer’s protocol (NEN Life Science Products). Vector infectivity was calculated as the ratio between titer and particle. At VIB IC and IDLV expressing FIX were produced by transient calcium-phosphate transfection of 293T cells as described previously (3). Cells were transiently transfected at 90% confluence using calcium-phosphate transfection (Invitrogen). Twenty-four hours after transfection, fresh D10 medium supplemented with Nu serum (BD Nu Serum IV) and 1.1 mg/ml ml Na-butyrate (Sigma, Belgium) was added, and viral vector-containing supernatant was collected at 24-hour intervals in two consecutive days and snap-frozen for later use. Non-concentrated vector batches were filtered on 0.45 mm filter (Corning, Elscolab, Belgium) and concentrated before *in vivo* injection using the Centricon Plus-70 filter system (Millipore) by centrifugation. Centrifugation was done for 90 minutes at 2000 rpm at 4°C. The retentate was retrieved by centrifuging for 5 minutes at 2000 rpm at 4°C. Concentrated vector aliquots were snap frozen and stored at -80°C for later use. IC and IDLV expressing FIX were titered using HIV-1 p24 core profile enzyme-linked immunosorbent assay (ELISA) (QuickTiter™ Lentivirus Quantitation Kit, Cellbiolabs).

***In Vitro* Experiments**

Huh7 cells were transduced with ICLV or IDLV expressing GFP (ET.GFP.142T) at multiplicity of infection (MOI) 50, 5, 0.5 and 3 days and 2 weeks after transduction GFP expression was analyzed by flow cytometry (BD Canto System) and total DNA was extracted using DNeasy Blood & Tissue Kit (Qiagen), according to manufacturer’s instructions. Human primary hepatocytes were purchased from Biopredic (France) according to a protocol approved by the San Raffaele Ethical Committee (TIGET-HPCT) and maintained following manufacturer’s instructions. Transduction with ICLV or IDLV was performed at different MOI (1 and 10). After 1 week of culture, nuclei were stained with Hoecsht 33258 (Sigma-Aldrich) and analyzed by live fluorescence microscopy; total DNA was extracted using DNeasy Blood & Tissue Kit (Qiagen), according to manufacturer’s instructions. Vector copy number (VCN) was determined using an *ad hoc* qPCR, which selectively amplifies the reverse transcribed vector genome (both integrated and non-integrated) discriminating it from plasmid carried over from the transient transfection (RT-LV; ΔU3 sense: 5’-tcactcccaacgaagacaagatc-3’, gag antisense: 5’ gagtcctgcgtcgagagag-3’). The amount of human DNA loaded in the reaction was quantified with a qPCR designed to amplify the hTERT gene as described (2).

**Cell preparation**

Mice were euthanized at the indicated time point, and spleens were collected and processed into single cell suspension. Splenic CD8+ T cells were magnetically isolated from splenocytes by negative selection kit (Miltenyi Biotec, Bergisch Gladbach, Germany). Intra-hepatic leukocytes (IHL), which include T cell infiltrates, were isolated from the liver by smashing the tissue and running the sample on a Percoll (Sigma) gradient, as previously described (4). Interferon-γ-secreting cells were enumerated by enzyme-linked immunospot (ELISPOT) assay in response to GFP-expressing cells as described (4).

**Flow cytometry**

Splenocytes and IHLs were stained with the following monoclonal antibodies (mAb): Allophicocyanin (APC) conjugated Foxp3 (Fjk-16s) staining kit (e-Bioscience); R- phycoerythrin (PE) conjugated anti-CD25 (PC61, Peridinin Chlorophyll (PerCP) conjugated anti-CD8a (53-6.7); Pacific Blue conjugated anti-CD4 (RM4-5) (BD Biosciences, Mountain View, CA). Detection of GFP-specific CD8+ T cells was performed by APC labeled Pro5 MHC pentamer H-2K^d^ HYLSTQSAL (GFP_200-208_) according to the manufactures instructions (Proimmune, Oxford, UK). Labeled cells were analyzed with a FACSCanto flow cytometer equipped with Diva software (BD Biosciences).

**Tissue analysis**

The liver was fixed in 4% paraformaldehyde, embedded in optimal cutting temperature (OCT), and frozen in iso-penthane pre-cooled in liquid nitrogen. Cryostat sections (10 µm thick) were blocked with 5% goat serum (Vector laboratories, Burlingame, CA), 1% bovine serum albumine (BSA), 0.1% Triton X-100 in PBS, and either directly analyzed under a 3-laser confocal microscope (Radiance 2100, Bio-Rad, Hercules, CA), or previously incubated with rabbit anti-GFP (Molecular Probe; Eugene, OR) washed and incubated with (FITC)-conjugated goat anti-rabbit immunoglobulin G. Sections form untreated mice were used as negative controls. Nuclei were stained with TOPRO-3 (Molecular Probe).

**Integration site analysis**

To analyze ICLV and IDLV integration in cultured cells, we used standard and non-restrictive 5’- and 3’-LTR mediated LAM-PCR as described (5, 6). Briefly, 100ng (for standard) or 500ng (for non-restrictive) DNA samples were pre-amplified by linear PCR using biotinylated primers hybridizing to LTR sequences: 5‘-TTAGCCAGAGAGCTCCCAGG-3‘ for 5’-LTR LAM-PCR and 5‘-AGCTTGCCTTGAGTGCTTCA-3‘ for 3’-LTR LAM-PCR. In combination with the previously reported linker cassette primers, two additional exponential amplifications were achieved using the following LTR primers, respectively: 5‘-GATCTGGTCTAACCAGAGAG-3‘ and 5‘-CCCAGTACAAGCAAAAAGCAG-3‘ for 5’-LTR LAM-PCR; 5‘-AGTAGTGTGTGCCCGTCTGT-3’ and 5‘-GATCCCTCAGACCCTTTTAGTC-3‘ for 3’-LTR LAM-PCR. Insertions of ICLV and IDLV in liver samples were analyzed by standard 3’LTR LAM-PCR with the primers previously described (5, 6). LAM-PCR amplicons were purified, tagged for downstream 454 pyrosequencing and bioinformatical analyses as previously described (7). Because of possible deletions in the LTR of the IDLV an amplicon sequence was considered as valid vector-host genome junction if the megaprimer sequence and at least 5 or 18 nucleotides of the LTR were present. The remaining sequences were automatically aligned to the mouse genome (assembly NCBI37/mm9) using BLAT (8). Alignments with at least 95% sequence identity were reported as integration sites. LAM-PCR amplicon sequences are available as open access database at https://consert.gatc-biotech.com/lampcr/. For username and password please contact the corresponding author MS

**Antigen Rechallenge**

DNA vaccination was performed 6 weeks after vector administration, as previously described (4). Briefly, 0.5 nmol cardiotoxin-1 (Sigma-Aldrich) was injected in triadic leg muscles. Five days later mice were injected again in the same position with 50 μg/leg of pCCLsin.cPPT.CMV.GFP.wpre plasmid. Mice were euthanized 12 days after DNA administration. Six months after vector administration mice were immunized with 4ug cFIX (Enzyme Research Laboratories) by means of subcutaneous injection (n=3 for both IC and IDLV). As control mice non-injected mice of the same strain (C57BL/6 FIX KO) were used (n=3). Canine FIX was solved in 100 ul PBS and was supplemented with 100 ul Incomplete Freund Adjuvant (IFA) (Sigma-Aldrich). As negative control (n=3, IDLV, ICLV and non-injected mice) mice were injected only with IFA. Immunization was repeated using exactly the same procedure a month later.

**Adoptive transfer**

C57Bl/6 OT-II TCR transgenic Ly5.2 mice were crossed with C57Bl/6 FOXP3-GFP knock-in Ly5.2 mice. FACS sorted Ly5.2 OT-II CD4^+^ ^FOXP3-GFP-^ were administred into C57Bl/6 Ly5.1 recipient mice by tail vein injection (2.5x10^6^ cells/mouse), one day before OVA-encoding IDLV or IDLV.142T administration. Reconstitution of Rag2^-/-^γ-chain^-/-^ mice was performed by intraperitoneal injection of pooled splenocytes and liver lymphocytes (6x10^7^ cells/mouse) isolated from naïve, IDLV-treated or IDLV-142T-treated as described above.

**B2-PCR and qPCR**

To check for genomic integration we use a PCR methods based on B2 repeats that are common in the mouse genome and that allow for amplification of integration junctions (9). For B2-PCR 2 primers sets were applied. Primer set 1: B2 sense: 5’-GGCTGGTGAGATGGTTCAGT-3’, 5NC2 antisense: 5’GAGTCCTGCGTCGAGAGAG-3’, corresponding nested primer set: LTR9 sense: 5’-GCCTCAATAAAGCTTGCCTTG-3’, U5PBS antisense:5’-GGCGCCACTGCTAGAGATTTT-3’; Primer set 2: WPRE sense: 5’-CTTTCCATGGCTGCTCGC-3’, B2 antisense: 5’-ATATGTAAGTACACTGTAGC-3’, corresponding nested primer set: Δnef sense: 5’-CGAGCTCGGTACCTTTAAGACC-3’; LTR8 antisense: 5’-TCCCAGGCTCAGATCTGGTCTAAC-3’. For amplication the follwing conditions were used: 5' B2-PCR 10 minutes at 95°C, 1 minute at 94°C, 1 minute at 57°C, 1 minute at 72°C, repeated 30 times, followed by an extra 7 minutes' extension at 72°C. The amplification conditions for the 3' B2-PCR were similar to the 5' B2-PCR but differed in that an annealing temperature of 49°C was used. A total volume of 50 µl was used containing 0.2 mM deoxynucleoside 5'-triphosphates, 1.5 mM MgCl2, 0.4 µM each of the primers, and 2.5 U Ampli Taq Gold (Perkin Elmer, Boston, MA). After this amplification, a nested PCR was performed with 2.5 µl B2-vector PCR product with 2 different internal primers in the vector genome. For the 5' nested PCR, the primers used were as follows: LTR9 sense, 5'-GCCTCAATAAAGCTTGCCTTG-3'; U5PBS antisense, 5'-GGCGCCACTGCTAGAGATTTT-3' amplifying a fragment of 121 bp. For the 3' nested PCR, the primers used were as follows: Delta nef sense, 5'-CGAGCTCGGTACCTTTAAGACC-3'; LTR8 antisense, 5'-TCCCAGGCTCAGATCTGGTCTAAC-3' amplifying a fragment of 166 bp. The amplification conditions were similar to the B2-vector PCR but differed in that the annealing temperature was 55°C for the 5' nested PCR and 58°C for the 3' nested PCR. As control, a nested PCR was performed with the use of 10 ng nonamplified genomic DNA from liver of treated and control mice. The PCR mix was subsequently subjected to 1.5% agarose gel electrophoresis. At VIB vector copy number (VCN) in the liver of mice injected with IC or IDLV expressing FIX was quantified by quantitative (q)PCR using an ABI7500 following the manufacturer’s instruction. Primers used for quantifying vector copy number were 5'-TGTGTGCCCGTCTGTTGTGT-3' and 5'-GAGTCCTGCGTCGAGAGAGC-3' and Taqman probe 5'-CGCCCGAACAGGGACTTGAA-3'. The qPCR reaction mix contains 12.5 µl of ABI-Q-PCR MASTER Mix, 0.75 µl of each 10 mM primers and 0.5 ml of the 10 mM Taqman Probe and 8.5 µl of gDNA containing 51 ng of total genomic DNA. Water was added to adjust the final volume. The qPCR reaction conditions were as follows: 50°C for 2 minutes, 95°C for 10 minutes, 40 cycles at 95°C for 15 seconds and 60°C for 1 minute. At TIGET vector DNA was quantified as follows: genomic DNA was extracted from liver samples by using “Maxwell 16 Tissue DNA Purification Kit” (Promega, Madison/WI, USA), according to manufacturer’s instructions. VCN were quantified by qPCR using a primer and probe set (LV sense: 5’-TACTGACGCTCTCGCACC-3’; LV antisense: 5’-TCTCGACGCAGGACTCG-3’, LV probe: FAM 5’-ATCTCTCTCCTTCTAGCCTC-3’) against the primer binding site (PBS) region of LV. Endogenous DNA amount was quantified by a primer/probe set against the human telomerase gene (Telo sense: 5’-GGCACACGTGGCTTTTCG-3’; Telo antisense: 5’- GGTGAACCTCGTAAGTTTATGCAA-3’; Telo probe: VIC 5’- TCAGGACGTCGAGTGGACACGGTG-3’ TAMRA) or the murine ß-actin gene (ß-Act sense: 5’-AGAGGGAAATCGTGCGTGAC-3’; ß-Act antisense: 5’-CAATAGTGATGACCTGGCCGT-3’; ß-Act probe: VIC 5’-CACTGCCGCATCCTCTTCCTCCC-3’). Copies per genome were calculated by the formula: (ng LV/ng endogenous DNA) x (n° of LV integrations in the standard curve). The standard curve was generated by using samples with previously determined copies by Southern blot analysis. All reactions were carried in out in triplicate in an ABI Prism 7900HT (Applied Biosystems, Foster City, CA).

**Inhibitors test**

Plasmas were screened for inhibitors with an activated partial thromboplastin time (aPTT) mixing assay, a standard screening assay for the presence or absence of inhibitory antibodies to either coagulation factor VIII or IX (10). Test plasmas were incubated in a 1:1 mix with normal plasma for 2 hours at 37 degrees and then the incubated mixture was assayed using standard aPTT reagents (11, 12). Control plasmas with known Bethesda Inhibitor titers of 2.6 (positive controls) and with no inhibitors (negative controls) were assayed concurrently for comparison.  In this manner, the animals that developed inhibitors were identified and that, when present, the inhibitor titer was higher than 2.6 BU.

**Image analysis and statistics**

Image pixel analysis to estimate the %GFP positive pixels and densitometry to assess the mean gray pixel value of the PCR bands was done by ImageJ (<http://rsb.info.nih.gov/ij/>). Statistical analysis was performed using Student t-test and ANOVA at α=0.05 level of confidence.

**References**

1. Follenzi A, Naldini L. HIV-based vectors. Preparation and use. Methods Mol Med 2002;69:259-274.

2. Lombardo A, Genovese P, Beausejour CM, Colleoni S, Lee YL, Kim KA, Ando D, et al. Gene editing in human stem cells using zinc finger nucleases and integrase-defective lentiviral vector delivery. Nat Biotechnol 2007;25:1298-1306.

3. Vandendriessche T, Thorrez L, Acosta-Sanchez A, Petrus I, Wang L, Ma L, L DEW, et al. Efficacy and safety of adeno-associated viral vectors based on serotype 8 and 9 vs. lentiviral vectors for hemophilia B gene therapy. J Thromb Haemost 2007;5:16-24.

4. Annoni A, Brown BD, Cantore A, Sergi LS, Naldini L, Roncarolo MG. In vivo delivery of a microRNA-regulated transgene induces antigen-specific regulatory T cells and promotes immunologic tolerance. Blood 2009;114:5152-5161.

5. Gabriel R, Eckenberg R, Paruzynski A, Bartholomae CC, Nowrouzi A, Arens A, Howe SJ, et al. Comprehensive genomic access to vector integration in clinical gene therapy. Nat Med 2009;15:1431-1436.

6. Schmidt M, Schwarzwaelder K, Bartholomae C, Zaoui K, Ball C, Pilz I, Braun S, et al. High-resolution insertion-site analysis by linear amplification-mediated PCR (LAM-PCR). Nat Methods 2007;4:1051-1057.

7. Cartier N, Hacein-Bey-Abina S, Bartholomae CC, Veres G, Schmidt M, Kutschera I, Vidaud M, et al. Hematopoietic stem cell gene therapy with a lentiviral vector in X-linked adrenoleukodystrophy. Science 2009;326:818-823.

8. Kent WJ. BLAT--the BLAST-like alignment tool. Genome Res 2002;12:656-664.

9. VandenDriessche T, Thorrez L, Naldini L, Follenzi A, Moons L, Berneman Z, Collen D, et al. Lentiviral vectors containing the human immunodeficiency virus type-1 central polypurine tract can efficiently transduce nondividing hepatocytes and antigen-presenting cells in vivo. Blood 2002;100:813-822.

10. Sahud, M.A. Factor VIII inhibitors. Laboratory diagnosis of inhibitors. Semin Thromb Hemost 2000;26:195-203.

11. Langdell, R.D., Wagner, R.H., and Brinkhous, K.M. Effect of antihemophilic factor on one-stage clotting tests. *J Lab Clin Med* 1953;41:637-647.

12. Nichols, T.C., Dillow, A.M., Franck, H.W., Merricks, E.P., Raymer, R.A., Bellinger, D.A., Arruda, V.R., et al. Protein replacement therapy and gene transfer in canine models of hemophilia A, hemophilia B, von Willebrand disease, and factor VII deficiency. ILAR J 2009;50:144-167.
